# Blood‐Lymphatic Integrated System with Heterogeneous Melanoma Spheroids via In‐Bath Three‐Dimensional Bioprinting for Modelling of Combinational Targeted Therapy

**DOI:** 10.1002/advs.202202093

**Published:** 2022-08-26

**Authors:** Won‐Woo Cho, Minjun Ahn, Byoung Soo Kim, Dong‐Woo Cho

**Affiliations:** ^1^ Department of Mechanical Engineering Pohang University of Science and Technology (POSTECH) Pohang Kyungbuk 37673 Republic of Korea; ^2^ School of Biomedical Convergence Engineering Pusan National University Yangsan Kyungnam 50612 Republic of Korea; ^3^ Institute for Convergence Research and Education in Advanced Technology Yonsei University Seoul 03722 Republic of Korea

**Keywords:** blood‐lymphatic integrated system, in‐bath bioprinting, intravasation, melanoma spheroids, metastatic melanoma

## Abstract

Although metastatic melanoma can be managed with chemotherapy, its heterogeneity and resistance to therapy remain poorly understood. In addition to the spread of melanoma in the bloodstream, melanoma‐stroma interaction and the lymphatic system play active roles in said heterogeneity and resistance, leading to its progression and metastasis. Reproducing the complexities of the melanoma microenvironment in vitro will help understanding its progression and enhance the translatability of potential cancer therapeutics. A blood‐lymphatic integrated system with heterogeneous melanoma spheroids (BLISH) using the in‐bath bioprinting process is developed. The process uniformly prints size‐controllable metastatic melanoma spheroids along with biomimetic blood and lymphatic vessels (LVs). The system reproduces hallmark events of metastatic melanoma, such as tumor stroma interaction, melanoma invasion, and intravasation. The application of the system to investigate the anticancer effect of combinational targeted therapy suggests that it can be used to study the pathophysiology of melanoma and improve the accuracy of drug response monitoring in skin cancer.

## Graphical abstract

Melanoma‐stroma interaction and the blood‐lymphatic system play active roles in melanoma progression and metastasis. In this study, a blood‐lymphatic integrated melanoma model via in‐bath bioprinting is developed. The system reproduces hallmark events of metastatic melanoma. The application of combinational targeted therapy suggests that the developed system can be used to study the pathophysiology of melanoma and improve the accuracy of drug response monitoring in skin cancer.

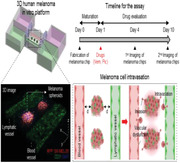

## 1 Introduction

Cutaneous melanoma, the deadliest form of skin cancer, has the highest mortality risk. The major cause of melanoma mortality originates from its metastasis to distant organs through the bloodstream and the lymphatic system. Treatment of metastatic melanoma is challenging because tumors rapidly develop resistance to conventional chemotherapy and retain carcinogenicity. Pathological data of melanoma patients reveal that melanoma is a complex tissue comprised of cancer cells and a dynamic combination of stromal cells.^[^ ^1^ ^]^ Therefore, researchers speculate that the mutual interactions between melanoma and stromal cells contribute to the progression and metastasis of melanoma.

In particular, the observation that most tumors are present as desmoplastic, palpable masses has caused studies to surmise that cancer‐associated fibroblasts (CAFs) are a major cellular component in tumor stroma.^[^ ^1^, ^2^ ^]^ Coculture experiments have indicated that CAFs assist the migration of cancer cells by generating microtracks for the invading cancer cells.^[^ ^3^ ^]^ Moreover, through the secretion of tumor‐promoting growth factors and extracellular matrix (ECM), CAFs form stromal niches and protect cancer cells from apoptosis foll(ecmowing treatment with chemotherapeutics.^[^ ^4^ ^]^ Due to this supportive microenvironment, cancer cells continuously proliferate and infiltrate the surrounding vasculature. In particular, during melanoma progression, lymphatic vessels (LVs) serve as a primary route for cancer cells to metastasize through the lymphatic system before systemically metastasizing through the blood vessel (BV).^[^ ^5^ ^]^ In clinical cases, regional lymph node metastasis is the most critical predictor of distant metastasis and death in melanoma.^[^ ^6^ ^]^ Therefore, cancer models that can reproduce the melanoma microenvironment with blood and lymphatic vasculature can prove a useful tool for studying the mechanisms of melanoma metastasis and testing the cellular response to anticancer drugs.

Preclinical animal models are considered the standard for studying cancer development. However, their clinical translatability to human cancer is debatable due to the fundamental mismatch of the physiological environments. The recent three‐dimensional (3D) cultured cancer models, especially spheroids, have become essential tools in cancer research as they emulate the structural complexity of in vivo solid tumors.^[^ ^7^ ^]^ Research on microfluidics‐based organ‐on‐a‐chip systems as vascularized cancer models has provided valuable insight into tumor‐vascular interaction and metastatic cascade.^[^ ^8^ ^]^ However, because of the spontaneous formation of vascular beds in organ‐on‐a‐chip systems, precise spatial control between tumor and vasculature is difficult, possibly affecting cell–cell interactions and the metastatic behavior of cancer cells.^[^ ^8b,8c^ ^]^ Moreover, scaling issues of the microfluidic system complicate the production and positioning of spheroids larger than 400 µm.^[^ ^9^ ^]^ These problems might limit the precise and consistent recapitulation of tumor interactions with the vasculature, including angiogenesis and melanoma metastasis.

3D bioprinting technology allows for programmable and precise deposition of printable hydrogels with cells, called bioink, to synthesize complex tissue constructs in vitro. Ideally, for melanoma cells to function properly, the bioink should resemble the structural and biochemical properties of the ECM. Schmid et al. developed a printable bioink to mimic the complex microenvironment of metastatic melanoma.^[^ ^10^ ^]^ However, for studying metastasis, researchers had to rely on the arteriovenous loop of the murine model that limited recapitulating tumor‐vascular interaction in human melanoma. Recent progress in bioprinting vascularized tissues has opened new avenues in developing advanced tumor‐vascular models. Meng et al. developed a metastatic cancer model for studying the key components of metastatic dissemination, including invasion, intravasation, and angiogenesis.^[^ ^8c^ ^]^ They used bioprinting to precisely locate the growth factor‐releasing capsules to induce cancer migration and angiogenesis. In a previous study, we studied the tumor‐vascular interactions occurring at proximal and distal sites through precise positioning of cellular components.^[^ ^8b^ ^]^ While these models successfully reproduced tumor‐vascular interactions and metastatic cascade to a large extent, the role of the lymphatic system, an essential feature in the development of in vitro cancer models, has been neglected. Recently, Cao et al. proposed a tumor‐on‐chip device with a bioprinted BV and LV pair.^[^ ^11^ ^]^ The developed platform reproduced the transport kinetics of drugs inside the tumor microenvironment (TME); however, the engineered acellular vessels could not accurately reproduce the melanoma microenvironment. In addition, tumor stroma interactions were poorly studied, possibly affecting future comprehensive studies on the progression and metastasis of melanoma.

In this study, we report an in‐bath bioprinting approach to engineer a BLISH. First, we fabricated a biomimetic skin tissue‐derived decellularized extracellular matrix (SdECM) bioink that allowed the direct bioprinting of numerous cellular components at precise locations. Moreover, the developed SdECM bioink retained a variety of growth factors and microstructure of ECM, thereby providing biological and physical microenvironments of native skin.^[^ ^12^ ^]^ Thus, we could print geometrically variable melanoma spheroids, reproducing the key features of tumor stroma within the SdECM bioink supporting bath, with a biomimetic BV and LV pair. The BLISH was used to recapitulate several key steps of metastatic dissemination. During maturation, heterogeneous melanoma spheroids exhibited BRAF inhibitor resistance by producing tumor stromal niches. Moreover, melanoma cells displayed relentless intravasation, disrupting the surrounding vasculature. As a proof of concept, we evaluated the anticancer effect of combinational cancer therapy to target melanoma and its stroma. Consequently, different combinations of inhibitors evoked different responses in melanoma progression and endothelial dysfunction in the BLISH. The developed in vitro 3D melanoma model with enhanced emulation of the complex TME should serve as an advanced platform for understanding the interaction of cancer cells with their microenvironment and screening latent anticancer drugs. The contributions of the study are as follows: 1) A method to bioprint metastatic cancer spheroids with a perfusable blood and lymphatic vessel pair; 2) A new inhibitor to suppress the metastasis of melanoma cells; 3) A system to simulate the steps in the spread and metastasis of melanoma from the bloodstream to the lymphatic system.

## 2 Results

### 2.1 In‐Bath Coaxial Bioprinting of Biomimetic Blood and Lymphatic Vessels

**Figure 1:**
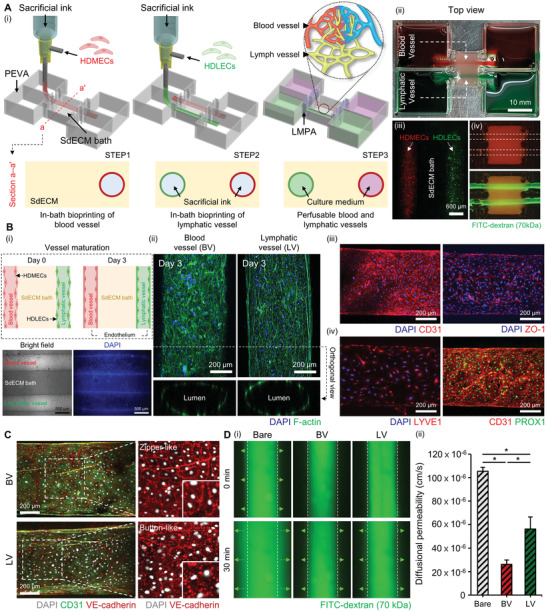
Engineering in vitro human BV and LV. Ai) Schematic of in‐bath coaxial bioprinting of biomimetic BV and LV. ii) Image illustrating the perfusion of color dye ink in each vessel. iii) Fluorescence images depicting bioprinted endothelial cells and (iv) perfusion of FITC‐dextran in each vessel. Bi) During the maturation of the printed vessels, proliferation of the HDMECs and HDLECs is observed through optical and nucleus staining. ii) 3D confocal images showing endothelium formation and maturation of the engineered vessels. iii) IF staining images of BV and iv) LV with tissue‐specific markers. C) Confocal images displaying the distribution of VE‐cadherin (red) in BV and LV. Magnified views of the areas in the white boxes indicate continuous zipper‐like junctions in the BV and discontinuous button‐like junctions in the LV. Di) Perfusion of FITC‐dextran in each vessel reveals ii) different diffusional permeability in each group. Error bars indicate the standard deviation (SD; *n* = 3). *p < 0.05 by one‐way ANOVA with Turkey's post hoc test.

We devised a new poly(ethylene‐vinyl acetate) (PEVA)‐based housing structure that contained the bath materials and enabled in‐bath bioprinting to engineer a BV and LV pair in vitro. We demonstrated the superior performance of coaxial bioprinting for the fabrication of vascular tissues with tunable geometry in a previous study.^[^ ^13^ ^]^ To verify its applicability for engineering the biomimetic BV and LV pair, we coaxially bioprinted perfusable channels using a hybrid bioink made of human dermal microvascular endothelial cells (HDMECs)‐ and human dermal lymphatic endothelial cells (HDLECs)‐encapsulated vascular tissue‐derived decellularized extracellular matrix (VdECM) (**Figure** **1**Ai). The fabrication process includes the following steps: 3D printing of a PEVA‐based housing structure, deposition of SdECM bath, bioprinting of each channel, and incubation at 37 °C for thermal crosslinking of the VdECM bioink in the shell part and dissolution of the sacrificial ink in the core part to produce a tubular structure. By alternately printing HDMECs and HDLECs along a straight path, we engineered two independent channels within the SdECM bath (Figure 1Aii). Fluorescence imaging revealed that the printed cells did not deviate from their initial printed location during crosslinking (Figure 1Aiii). The channels were quickly filled with fluorescein isothiocyanate (FITC)‐dextran (70 kDa) solution from one side to verify the perfusability of each channel (Figure 1Aiv).

Next, we verified maturation and endothelium formation in each vessel after printing. Optical and fluorescence imaging revealed a confluence of endothelial cells in each vessel on day 3 of the culture (Figure 1Bi). Moreover, the confluent expression of filamentous actin (F‐actin) was observed in the printed BV and the LV owing to the supportive microenvironment of the VdECM hybrid bioink and the surrounding SdECM bath (Figure 1Bii). Interestingly, compared with the monoculture condition, LV cocultured with BV exhibited higher cell viability, probably because of the continuous nutrient diffusion from BV and cellular crosstalk between each vessel (Figure S1, Supporting Information). Subsequently, we conducted the immunofluorescence (IF) staining of BV and LV with vascular and lymphatic tissue‐specific markers to observe the recapitulation of native phenotypes of BV and LV. The staining of CD31 and tight junction marker zonula occludens‐1 (ZO‐1) revealed that BV underwent successful endothelialization (Figure 1Biii). The LV phenotype was verified by IF staining LV endothelial receptor 1 (LYVE1) and prospero homeobox protein 1 (PROX1) (Figure 1Biv), known as mature lymphatic markers.^[^ ^14^ ^]^

The BV and LV display significantly different patterns in the intracellular adherens junction.^[^ ^15^ ^]^ While blood capillaries have a continuous zipper‐like junction (associated with low permeability), lymphatic capillaries have a discontinuous junction, called a button‐like junction. This button‐like junction makes the lymphatic capillaries more permeable and works as a primary valve, regulating the drainage of interstitial fluid and immune cell entrance into the LV.^[^ ^16^ ^]^ The distribution of adherens junctions in BV and LV were examined by IF‐staining VE‐cadherin. VE‐cadherin was continuously expressed in the BV, forming a zipper‐like tight junction. In contrast, the LV exhibited the formation of a discontinuous, button‐like junction (Figure 1C). The diffusional permeability of the channels is directly related to the barrier function, which is regulated by the adherens junctions between the endothelial cells. Therefore, FITC‐dextran solution was perfused in each channel to evaluate the influence of this junctional difference on diffusional permeability. The diffusional permeability of the channel without endothelial cells (bare), BV, and LV was examined through time‐lapse fluorescence imaging (Figure 1Di). On the one hand, while the dextran solution rapidly expanded in the bare channel (105.39 × 10^–6^ cm s^−1^), the solution was contained within the channel in the BV, owing to the intercellular junction of HDMECs (26.14 × 10^–6^ cm s^−1^) (Figure 1Dii). On the other hand, the LV displayed a higher diffusional permeability (56.25 × 10^–6^ cm s^−1^) than the BV, possibly attributed to the permeable characteristic of the button‐like junction between HDLECs.

### 2.2 In‐Bath Bioprinting of Metastatic Melanoma Spheroids

**Figure 2:**
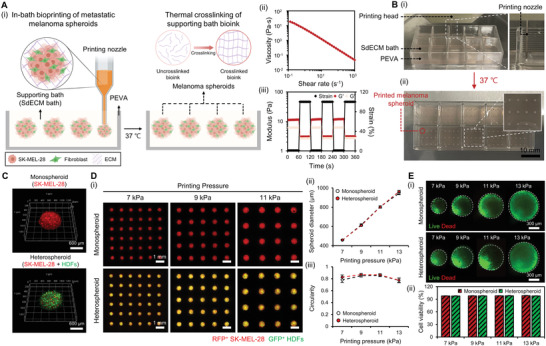
In‐bath bioprinting of metastatic melanoma spheroids within the SdECM bioink supporting bath. Ai) Entire schematic of in‐bath bioprinting of metastatic melanoma spheroids within the SdECM bath. ii) Shear‐thinning behavior and iii) Shear recovery of SdECM bioink according to the changes in strain. Bi) Images illustrating the bioprinting process and ii) Printed melanoma spheroids after crosslinking at 37 °C. C) 3D confocal images of the monospheroid and heterospheroid after printing within the SdECM bath. Di) Precise size control of the spheroids by varying the printing pressure. ii) Corresponding graphs for the measured spheroid diameter and iii) circularity of the printed spheroids (*n* = 36). Ei) Live/dead staining of monospheroids and heterospheroids after printing at different printing pressures. ii) Quantified cell viability of the printed spheroids along the printing pressure (*n* = 3). Error bars indicate the SD.

We developed a method for bioprinting the mixture of melanoma and stromal cells as spheroid within the supporting bath material to enable in‐bath bioprinting of metastatic melanoma spheroids. After sequentially printing the PEVA housing structure and SdECM bioink supporting bath, we printed the cells‐laden SdECM bioink as spheroids (**Figure** **2**Ai). The printed spheroids were incubated in a CO_2_ incubator at 37 °C to induce thermal crosslinking of the SdECM bioink. Therefore, the printed cells were allowed to preserve their spheroidal shape through continued culturing. To apply this method, the supporting bath material should exhibit shear‐thinning and self‐healing for stable localization of the spheroids in the right position after the printing process. In previous studies, we demonstrated the performance of the SdECM bioink in recapitulating tissue‐specific microenvironment and engineering skin tissues.^[^ ^12^, ^17^ ^]^ We performed a rheological analysis on 0.75% (w/v) SdECM bioink to verify its potential as a supporting bath material for in‐bath bioprinting. Consequently, the bioink displayed shear‐thinning behavior in the established shear rate range (Figure 2Aii). In addition, the shear recovery assessment indicated the recovery of storage modulus during cycles of high to low strains (Figure 2Aiii). Combining the rheological results, SdECM bioink could serve as a supporting bath for in‐bath bioprinting of melanoma spheroids, confirmed by using in‐bath bioprinting for the direct and precise positioning of the metastatic melanoma spheroids (Figure 2Bi). After printing the spheroids, the printed structure was transferred to an incubator to induce thermal crosslinking of the SdECM bath. The resulting image confirmed that the original shape and position of the printed spheroids were preserved within the SdECM bath after crosslinking (Figure 2Bii).

Using this approach, we prepared two experimental groups of melanoma spheroids: monospheroid (SK‐MEL‐28 only) and heterospheroid (SK‐MEL‐28 and dermal fibroblasts). Among the various metastatic melanoma cell lines (Figure S2, Supporting Information), we printed the monospheroids made of SK‐MEL‐28, a human malignant melanoma cell line expressing BRAF(V600E) mutated form, due to their high invasiveness and high suitability for studying metastatic melanoma.^[^ ^7^, ^18^ ^]^ The heterospheroids were synthesized by integrating the human dermal fibroblasts, which play a predominant role in melanoma progression. 3D confocal images revealed that both monospheroids and heterospheroids were stably printed while conserving the spheroidal shape within the SdECM bath (Figure 2C). Having demonstrated the ability of the system to print metastatic melanoma spheroids within the SdECM bath, we now assess the influence of printing pressure on the diameter of the printed spheroid. Spheroids of different sizes were printed by increasing the printing pressure from 7 to 13 kPa at a fixed printing time (300 ms per spheroid) and a needle gauge of 26G (Figure 2Di). The diameters of the spheroids were measured from 460 to 950 µm at pressures of 7, 9, 11, and 13 kPa (Figure 2Dii). The measured diameter of the spheroids increased linearly with the printing pressure. We observed that both the printed monospheroids and heterospheroids had similar sizes at each printing pressure, suggesting the dominant influence of printing pressure on the size of the spheroids. However, the roundness of the printed spheroids was maintained at ≈0.8, irrespective of the printing pressure (Figure 2Diii). This roundness is similar to that of the conventional spheroids.^[^ ^19^ ^]^ Subsequently, live/dead staining was conducted to investigate the effect of printing pressure on cell viability. Fluorescence images revealed that most cells were alive after printing, regardless of the printing pressure (Figure 2Ei). Live/dead staining confirmed that the cell viability of the printed spheroids was above 95% in the pressure range (Figure 2Eii).

These results confirm that the in‐bath bioprinting technology enables the precise size control and repetitive positioning of metastatic melanoma spheroids within the SdECM bath. Similar to the microwell arrays or microfluidic‐based fabrication methods, the size of the spheroids in this method was easily controlled by adjusting the printing pressure.^[^ ^19^, ^20^ ^]^ The experimental results showed that the in‐bath bioprinting technology can be used to produce micro‐sized multicellular cancer spheroids of ≈400–900 µm directly within the ECM‐based bioink by controlling the printing pressure. We observed that the diameter of the printed spheroids was strongly influenced by the printing pressure and was unaffected by the type of cell.

### 2.3 Fibroblasts‐Assisted Melanoma Invasion and Formation of Tumor Stromal Niches

**Figure 3:**
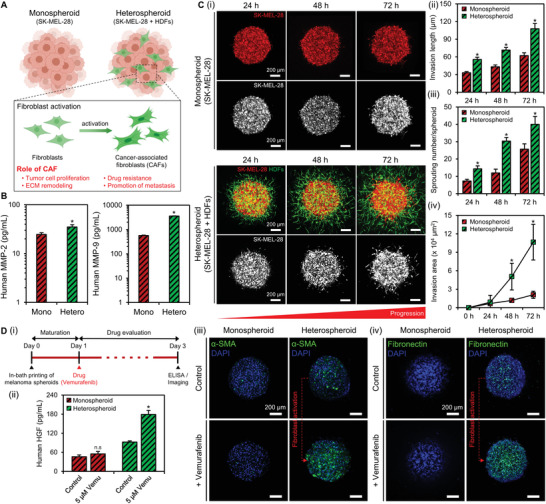
Fibroblast‐assisted melanoma progression and formation of tumor stromal niche. A) Fibroblast activation in melanoma and the role of the activated fibroblasts in the TME. B) Measurement of MMP‐2 and MMP‐9 secretion by the melanoma spheroids on day 3. Ci) Sprouting of the monospheroids and heterospheroids within the SdECM bath over 72 h. White fluorescence images represent spouting of the melanoma cells. ii) Quantification data of the invasion length, iii) the sprouting number, and iv) invasion area of the melanoma cells over 72 h. Di) Timeline for studying the progression of melanoma in response to vemurafenib treatment. ii) Measurement of HGF secretion by the melanoma spheroids on day 3. iii) Expression of *α*‐SMA and iv) fibronectin is evaluated in the melanoma spheroids. The experiments were repeated three times with triple replications. Error bars indicate the SD (*n* = 3). *p < 0.05 by a two‐tailed Student's *t*‐test.

The interaction of melanoma cells with the dermal fibroblasts is pivotal to the progression and metastatic growth of melanoma.^[^ ^21^ ^]^ During metastatic progression, surrounding fibroblasts are activated through crosstalk with the melanoma cells through paracrine signaling and differentiated into CAFs. The CAFs subsequently acquire the properties of myofibroblasts and produce various growth factors, cytokines, and ECM, thus remodeling the surrounding matrix and creating a favorable niche for the melanoma to grow, invade, and gain drug resistance (**Figure** **3**A). The paracrine effect of the printed melanoma spheroids on the activation of dermal fibroblasts was evaluated through the transwell coculture system to recapitulate the activation of fibroblasts in the TME (Figure S3, Supporting Information). As per the IF staining results, fibroblasts cocultured with the melanoma spheroids (coculture) formed thick actin stress fibers, exhibiting an increased expression of alpha‐smooth muscle actin (*α*‐SMA) and the stromal marker vimentin (Figure S4A, Supporting Information). The results of the quantitative reverse transcription‐polymerase chain reaction (qRT‐PCR) analysis indicated that the expression of CAFs‐related markers significantly increased in the cocultured fibroblasts, consistent with the IF staining results (Figure S4B, Supporting Information). The results of the wound‐healing assay of fibroblasts showed that the coculture group (81.8 ± 1.4%) had a remarkably larger recovered scratch area than the monoculture group (56.5 ± 7.7%), indicating enhanced fibroblasts motility in the coculture group (Figure S5, Supporting Information).

Metastatic melanoma is characterized by high levels of matrix metalloproteinase (MMP)‐2 and ‐9 secretions that decompose the surrounding ECM, allowing melanoma cells to spread to other organs.^[^ ^22^ ^]^ Therefore, to investigate the release level of MMPs by the printed spheroids, the amounts of MMP‐2 and MMP‐9 were analyzed through enzyme‐linked immunosorbent assay (ELISA). Consequently, the heterospheroid (35.1 ± 3.8 pg Ml^−1^) secreted more MMP‐2 than the monospheroid (24.7 ± 2.0 pg mL^−1^) (Figure 3B). Notably, the heterospheroid (3574 .5 ± 39.6 pg mL^−1^) secreted significantly more MMP‐9 than the monospheroid (564.5 ± 25.9 pg mL^−1^). Additionally, we measured the amount of MMP‐2 (25.9 ± 3.1 pg mL^−1^) and MMP‐9 secretion (1236.2 ± 30.9 pg mL^−1^) in the fibroblasts monospheroid group to evaluate if the increased MMP secretion in the heterospheroid group depended solely on the incorporation of fibroblasts (Figure S6, Supporting Information). The results confirmed that MMP secretion was highest in the heterospheroid group. These observations indicate that the interaction between melanoma cells and the fibroblasts increased secretion of MMPs in the heterospheroid group, consequently resulting in enhanced sprouting and melanoma invasion into the surrounding matrix.

We conducted a 3D spheroid invasion assay to examine the effect of the CAFs on melanoma invasion. After in‐bath bioprinting, the melanoma spheroids were cultured for three days to observe metastatic behavior within the SdECM bath. During the in vitro culture, we observed the sprouting of the melanoma cells in both groups (Figure 3Ci). The data demonstrated significantly increased sprouting in the heterospheroid compared with the monospheroid. SK‐MEL‐28 in the heterospheroid group had a significantly longer invasion length for 72 h (107.8 ± 8.9 µm) than that in the monospheroid group (62.1 ± 4.8 µm) (Figure 3Cii). In addition to the invasion length, the sprouting number of SK‐MEL‐28 was also higher in the heterospheroid (40 ± 4.5 per spheroid) than in the monospheroid (19.3 ± 2.9 per spheroid) (Figure 3Ciii)). Likewise, melanoma cells in the heterospheroid had a larger total invasion area (10.6 ± 3.1 × 10^4^ µm^2^) than that in the monospheroid (2.1 ± 0.6 × 10^4^ µm^2^) (Figure 3C (iv)). The gap in the invasion area between each group further increased over 72 h. Labernadie et al. demonstrated that CAFs assisted cancer progression by heterophilic adhesion of N‐cadherin at the CAF membrane and E‐cadherin at the cancer cell membrane.^[^ ^3b^ ^]^ Correlating with this result, melanoma cells in the heterospheroid tend to follow the migration path of the preceding fibroblasts, suggesting fibroblast‐assisted melanoma migration and invasion (Figure S7, Supporting Information).

Several studies suggest that a fibroblast‐derived niche can offer the therapeutic escape of melanoma cells from BRAF inhibition.^[^ ^2^, ^23^ ^]^ Reports also suggest that the BRAF inhibitor itself plays a critical role in activating fibroblasts in the tumor tissue. With the hepatocyte growth factor (HGF) signaling and production ECM, CAFs form a protective niche by producing fibronectin for cancer cell adhesion to protect cancer cells from apoptosis following treatment with the BRAF inhibitor.^[^ ^24^ ^]^ To recapitulate these phenomena in the proposed model, we applied vemurafenib (a small‐molecule inhibitor of BRAF(V600E) kinase approved by the Food and Drug Administration (FDA) for the treatment of BRAF‐mutant melanoma) to the printed melanoma spheroids (Figure 3Di). The role of the vemurafenib‐activated fibroblasts in the release of HGF was demonstrated by the ELISA (Figure 3D (ii)). The results demonstrate that administering vemurafenib significantly increased the secretion of HGF in the heterospheroids (≈2 times), while no significant difference was observed in the monospheroids (≈1.1 times). *α*‐SMA staining revealed that administering vemurafenib increased the expression of *α*‐SMA in the heterospheroids, indicating BRAF‐inhibition‐induced fibroblasts activation (Figure 3Diii). Moreover, IF staining revealed an increased deposition of fibronectin by the activated fibroblasts in the vemurafenib‐treated heterospheroids. Previous studies had reported that the adhesion of cancer cells to fibronectin facilitated carcinogenesis and induced resistance to chemotherapeutic agents.^[^ ^25^ ^]^ In the heterospheroids, the administration of the BRAF inhibitor increased fibroblast activation and induced the formation of tumor stromal niche, whereas, in the monospheroids, no fibronectin formation was observed regardless of treatment with vemurafenib (Figure 3Div). Based on these results, we conducted the following experiments using heterospheroids for the better recapitulation of tumor stroma interaction in the TME.

### 2.4 Evaluation of Combinational Treatment of BRAF Inhibitor with PI3K Inhibitor

**Figure 4:**
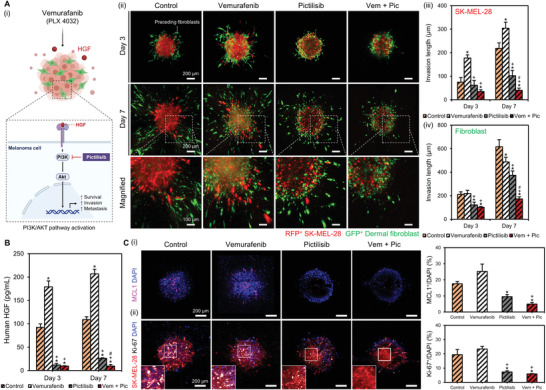
Effect of the BRAF/PI3K combined inhibitor on melanoma repression. Ai) BRAF/PI3K combined inhibitor prevents HGF‐mediated PI3K/AKT pathway activation in melanoma. ii) Representative 3D confocal images illustrating the local invasion of the melanoma spheroids, following treatment with 5 µM vemurafenib, 5 µM pictilisib, or their combination for 3 and 7 days. iii) Quantification data of invasion length of the melanoma cells and iv) the fibroblasts over 7 days. B) ELISA depicting the release of HGF from the heterospheroids, following treatment with 5 µM vemurafenib, 5 µM pictilisib, or their combination. Ci) IF staining images and quantification data of MCL1 and (ii) Ki‐67 expression in each group. The experiments were repeated three times with triple replications. Error bars indicate the SD (*n* = 3). *p*‐values are calculated using one‐way ANOVA with Turkey's post hoc test. *p < 0.05 indicates the significance between the control group and the rest. +*p* < 0.05 shows the significance between the vemurafenib group and the rest. #*p* < 0.05 shows the significance between the pictilisib and vem + pic groups.

The phosphatidylinositol 3‐kinase (PI3K)/protein kinase B (AKT) signaling pathway is a key regulator of multiple cellular physiological processes, including cell cycle, growth, proliferation, survival, and apoptosis.^[^ ^26^ ^]^ Research demonstrated that the augmented activation of the PI3K/AKT pathway contributed to the survival and proliferation of cancer cells in several types of human cancer.^[^ ^27^ ^]^ In melanoma, the adhesion of melanoma cells to fibronectin is critical in amplifying the HGF‐mediated PI3K/AKT pathway after the treatment of BRAF inhibitor (**Figure** **4**Ai). Therefore, we assessed the efficacy of BRAF/PI3K combined inhibition in inhibiting melanoma progression in the tumor stromal niche and enhancing the antitumor effect of vemurafenib. The inhibitory effect of the combinational treatment of vemurafenib with a PI3K inhibitor, pictilisib, on the melanoma spheroids was assessed for the following experimental groups: melanoma cultured with normal growth medium (control), 5 µM vemurafenib (vemurafenib), 5 µM pictilisib (pictilisib), or their combination (vem + pic). Consequently, we observed significantly different migration behaviors in the melanoma cells and fibroblasts over seven days of culture (Figure 4Aii). On the one hand, melanoma cells in the vemurafenib group exhibited more invasiveness (304.3 ± 25.7 µm) than the cells in the control group (218.6 ± 23.2 µm), possibly attributed to the vemurafenib‐induced fibroblasts activation (Figure 4Aiii). On the other hand, melanoma invasion in the vem + pic group was effectively suppressed (40.1 ± 9.6 µm), suggesting the synergistic effect of BRAF/PI3K combined inhibition. Additionally, fibroblasts in the vem + pic group demonstrated the shortest invasion distance (172.5 ± 18.2 µm), indicating that combinational therapy effectively inhibited the activation of fibroblasts as well as melanoma cells (Figure 4Aiv).

Based on the results from the spheroid migration assay, we examined the effect of BRAF/PI3K combined inhibition on HGF secretion in the heterospheroids through ELISA. In line with the sprouting assay results, the vemurafenib‐treated group had the largest quantity of HGF secretion on day 7 (206.9 ± 9.8 pg mL^−1^), compared with the control group (108.9 ± 6.2 pg mL^−1^) and the pictilisib group (26.2 ± 1.3 pg mL^−1^) (Figure 4B). Interestingly, the amount of HGF detected in the vem + pic group (11.4 ± 0.9 pg mL^−1^) significantly decreased by the cancer‐inhibiting effect of the BRAF/PI3K inhibitor. Moreover, the vem + pic group showed the weakest expression of myeloid leukemia 1 (MCL‐1) (Figure 4Ci), an antiapoptotic protein of B‐cell lymphoma 2 (BCL‐2) family involved in tumorigenesis, poor prognosis, and drug resistance by binding to the pro‐apoptotic BCL‐2 proteins.^[^ ^28^ ^]^ The expression of Ki‐67, a representative marker of cancer cell proliferation, was also significantly reduced in the vem + pic group, indicating the diminished proliferation of melanoma cells in the group (Figure 4Cii). Collectively, these results demonstrate that the combination of BRAF and PI3K inhibitors effectively reduced vemurafenib‐induced fibroblast activation and exhibited notable efficacy in suppressing melanoma invasion and proliferation.

### 2.5 Effect of BRAF/PI3K Combined Inhibitor on Melanoma Intravasation and Endothelial Disruption

**Figure 5:**
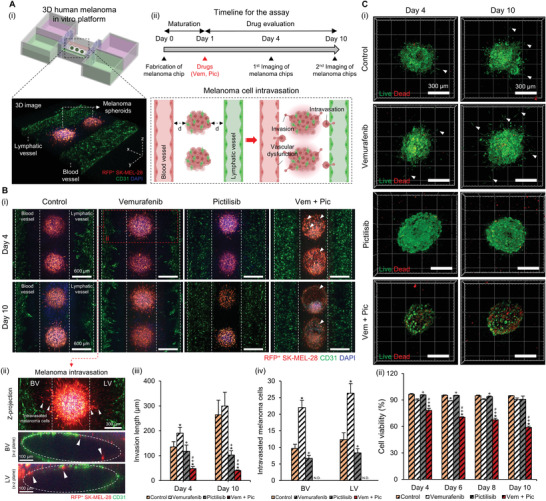
Anticancer effect of BRAF/PI3K combined inhibitor. Ai) Developed 3D human melanoma in vitro platform comprises melanoma spheroids, BV, and LV. ii) Timeline for the observation of the progression and intravasation of melanoma in response to BRAF/PI3K combined inhibitor. Bi) Representative 3D confocal images of the melanoma platform, following treatment with 5 µM vemurafenib, 5 µM pictilisib, or their combination. In the vem + pic group, melanoma spheroids begin to exhibit a distorted morphology after drug administration (white arrow). ii) Representative cross‐section images of BV and LV illustrating transendothelial migration of the melanoma cells (white arrow). iii) Quantification data of the invasion length, and iv) the number of intravasated melanoma cells over 10 days. Ci) Live/dead staining images and ii) quantified cell viability of the melanoma spheroids after drug administration. White arrows indicate sprouting of the melanoma spheroids. The experiments were repeated three times with triple replications. Error bars indicate the SD (*n* = 3). *p*‐values are calculated using one‐way ANOVA with Turkey's post hoc test. *p < 0.05 shows the significance between the control group and the rest. +*p* < 0.05 shows the significance between the vemurafenib group and the rest. #*p* < 0.05 shows the significance between the pictilisib and vem + pic groups.

Intravasation is an initial step in metastasis where cancer cells transmigrate into the lumen of BV or LV and enter the circulatory system, thereby becoming circulating tumor cells.^[^ ^29^ ^]^ We demonstrated the effective suppression of fibroblast‐assisted melanoma invasion and proliferation of melanoma cells by combining BRAF/PI3K inhibitors. We engineered the BLISH, which included metastatic melanoma spheroids with a perfusable BV and LV pair, to evaluate the effect of administration of the BRAF and PI3K inhibitors on melanoma intravasation (**Figure** **5**Ai). Considering the versatility of the in‐bath bioprinting technology, melanoma spheroids were precisely positioned in the middle of the vessels. In the BLISH, we focused on recapitulating the hallmark events of melanoma metastasis and evaluating the anti‐metastatic effect of BRAF/PI3K combined inhibition, as well as investigating the tumor‐induced endothelial disruption. During the in vitro culture, drugs were administrated through the BV to emulate blood‐flow‐driven drug delivery in vivo, and the metastatic behavior of melanoma cells was investigated according to the timeline (Figure 5Aii).

Consequently, we observed significant differences in melanoma invasion toward the peripheral BVs and LVs within each experimental group. The results of confocal imaging revealed a remarkable decrease of the sprouting of SK‐MEL‐28 in the vem + pic group (Figure 5Bi). Interestingly, morphologic distortion of the melanoma spheroids was observed in the vem + pic group on day 4. The spheroids were severely destroyed on day 10, caused by drug‐induced cancer cell apoptosis. The apoptosis of the melanoma spheroids in the vem + pic group was verified through optical imaging (Figure S8, Supporting Information). In other groups, however, melanoma cells relentlessly invaded the surrounding matrix and even adhered to the endothelium of the surrounding vessels. The orthogonal images of the vemurafenib group in Figure 5Bii display the intravasation of the melanoma cells, where the RFP‐tagged melanoma cells transmigrate the luminal surface of both BV and LV. The quantification data revealed that the invasion length of the melanoma cells was highest in the vemurafenib group (299.5 ± 54.9 µm), followed by the control (264.3 ± 58.5 µm), pictilisib (102.6 ± 17.1 µm), and vem + pic groups (40.1 ± 2.7 µm) (Figure 5Biii). Next, we calculated the number of intravasated melanoma cells in each group (Figure 5Biv). For BVs, the vemurafenib‐treated group had the highest number of intravasated melanoma cells (22 ± 2.2 cells per vessel). Similarly, for the LV, the incidence of melanoma intravasation in the vemurafenib group (26.3 ± 2.9 cells per vessel) was significantly higher compared with that in the other groups, indicating increased melanoma invasion mediated by the activated fibroblasts. Interestingly, intravasation of the melanoma cells was not detected in the vem + pic group, suggesting that the BRAF/PI3K combined inhibition effectively suppressed the sprouting of those cells, thus preventing metastasis. The cell viability of the melanoma spheroids was then assessed using live/dead staining, to evaluate the anticancer effect of the drugs (Figure 5Ci). We observed that the viability of the melanoma spheroids in the vem + pic group decreased over time (58.9 ± 0.5% at day 10), while the others preserved cell viability of more than 90% (Figure 5Cii).

**Figure 6:**
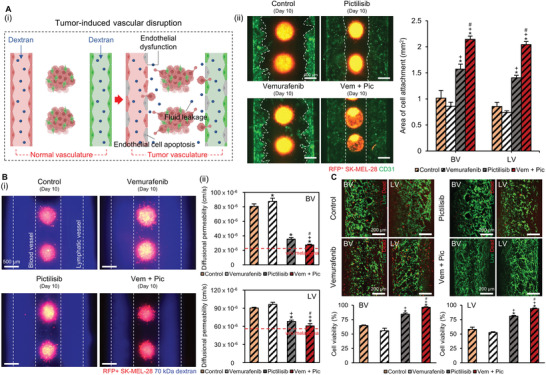
Effect of the BRAF/PI3K combined inhibitor on endothelial dysfunction. Ai) The engineered platform is utilized to study tumor‐induced vascular disruption (e.g., endothelial dysfunction, fluid leakage, endothelial cell apoptosis) in the tumor vasculature. ii) Fluorescence imaging is used to evaluate melanoma‐induced endothelial detachment in each group. Bi) Perfusion of FITC‐dextran reveals reduced diffusion of macromolecules in the vem + pic group compared with the rest. ii) Significant increase in the diffusional permeability is observed in both BV and LV, except for the vem + pic group. C) Live/dead staining to assess the tumor‐induced endothelial cell apoptosis in BV and LV. Error bars indicate the SD (*n* = 3). *p*‐values are calculated using one‐way ANOVA with Turkey's post hoc test. *p < 0.05 shows the significance between the control group and the rest. +*p* < 0.05 shows the significance between the vemurafenib group and the rest. #*p* < 0.05 shows the significance between the pictilisib and vem + pic groups.

In the TME, tumor vasculature is morphologically unstable because of the structural deformations induced by MMP secretion, paracrine signaling, and tumor–endothelium‐cell contact.^[^ ^30^ ^]^ This instability causes endothelial disruption or apoptosis of the endothelial cells, disrupting the tumor vasculature and vulnerating it to invasion by the cancer cells (**Figure** **6**Ai). Therefore, following cancer metastasis, we studied the disruption of BV and LV induced by the melanoma spheroids. We conducted fluorescence imaging to observe the effects of melanoma spheroids on endothelial destruction. Consequently, the vessels were distorted, and the endothelial cells were detached severely in the control and vemurafenib‐treated groups, owing to the relentless proliferation and metastasis of the cancer cells (Figure 6Aii). In the vem + pic group, the BV and LV preserved their original shape without undergoing significant distortions. Next, we verified the permeability of the vessels in each group to assess the fluid leakage from endothelial disruption. Also, the BV and LV with monospheroids made of melanocytes, melanoma cells, or fibroblasts were prepared to compare the effect of MMPs‐producing fibroblasts on diffusional permeability in each group (Figure S9, Supporting Information). In this study, BV and LV cocultured with fibroblasts spheroids showed a significant increase in diffusional permeability, probably due to the MMP‐producing fibroblasts. Furthermore, BV and LV in the control and vemurafenib groups exhibited a critical increase in the diffusional permeability compared to those cocultured with fibroblasts spheroids due to the increased MMP secretion result from tumor‐stroma interaction in the heterospheroids (Figure 6B (i)). BV (27.47 × 10^–6^ cm s^−1^) and LV (60.83 × 10^–6^ cm s^−1^) in the vem + pic group had a permeability value similar to those of BV (26.14 × 10^–6^ cm/s) and LV (56.25 × 10^–6^ cm s^−1^) without the melanoma spheroids (Figure 6Bii). Next, we conducted live/dead staining in each group to assess the tumor‐induced endothelial cell apoptosis (Figure 6C). Similar to the previous results, BV (96.3 ± 1.1%) and LV (93.9 ± 1.2%) in the vem + pic group were notably more viable than those in other groups, owing to the effective suppression of melanoma invasion and proliferation by BRAF/PI3K combined inhibition.

## 3 Conclusion

We used the in‐bath bioprinting process to fabricate in one step size‐controllable metastatic melanoma spheroids with a biomimetic BV and LV pair. The BLISH enabled multi‐pronged analysis of melanoma metastasis and tumor‐induced endothelial dysfunction in response to combination targeted therapy. The BRAF/PI3K inhibitor effectively suppressed CAF‐mediated drug resistance and decreased cancer invasion. In addition, tumor‐induced endothelial dysfunction was significantly reduced, suggesting the synergistic anticancer effect of the BRAF/PI3K inhibitor. The results suggest that the BLISH has potential applications in the study of cancer metastasis and enhancement of accuracy in monitoring drug responses based on the interaction of cancer cells and the surrounding TME. According to our research, this is the first study enabling the bioprinting of metastatic cancer spheroids with perfusable BV and LV pair. The limitation of this study is the lack of diversity of the metastatic cancer cell types (e.g., lung, breast, brain, etc.). However, the platform still provides a convenient and effective way for screening drug candidates and evaluating drug efficacy. Due to the versatility of in‐bath bioprinting, future studies on various metastatic cancers can introduce current cellular components and tissue‐specific bioinks. Furthermore, we believe that adopting patient‐derived cell populations or organoids will offer a pathway to developing personalized cancer models for application in precision medicine. Another limitation of the study is that that incorporating small vessels and microvessels are still challenging through our in‐bath bioprinting technology. Recently, Son et al. reported a novel bioprinting technology for the fabrication of multiscale microvasculature, which can be combined with our methodology in the future.^[^ ^31^ ^]^ Additionally, introducing a growth factor gradient to induce angiogenesis may be required to fabricate a multiscale network of blood and lymphatic vessels, enabling a more profound understanding of the interaction between cancer cells and the surrounding vasculature.^[^ ^32^ ^]^ The geometry of the tumor is another critical factor that affects cancer development and metastasis.^[^ ^33^ ^]^ Through in‐bath bioprinting technology, we could easily control the diameter of the melanoma spheroids. In the future, it might be necessary to incorporate this concept into our research to study the effect of the geometrical cues on cancer invasion and tumorigenicity.

## 4 Experimental Section

### Cell Source and Culture

Malme‐3 M, SK‐MEL‐2, and SK‐MEL‐28 cells (malignant melanoma cell line from ATCC) were maintained in the RPMI 1640 medium (Gibco), supplemented with 10% fetal bovine serum (FBS, Gibco) and 1% Pen‐Strep (Gibco). A375‐P cells (ATCC) and adult human dermal fibroblasts (NHDF‐Ad, Lonza) were cultured in Dulbecco's modified eagle medium with high glucose (DMEM, Cytiva), supplemented with 10% FBS and 1% Pen‐Strep. Adult human dermal microvascular endothelial cells (HDMECs, PromoCell) were cultured in the endothelial cell growth medium MV (EGM‐MV, PromoCell), supplemented with 1% Pen‐Strep up to passage 4. Adult human dermal lymphatic endothelial cells (HDLECs, PromoCell) were cultured in the endothelial cell growth medium MV2 (EGM‐MV2, PromoCell), supplemented with 1% Pen‐Strep up to passage 5. Normal human epidermal melanocytes (NHEMs, PromoCell) were cultured in melanocyte growth medium, supplemented with 1% Pen‐Strep up to passage 7.

### Evaluation of Rheological Properties of SdECM Bioink

The rheological properties of SdECM bioink were quantified using a rheometer (TA Instruments) with a 25 mm diameter plate geometry. We performed steady shear sweep analysis of SdECM bioink with a concentration of 0.75% (w/v) at a constant temperature of 15 °C to assess the shear‐thinning effect. The shear recovery behavior of the SdECM bioink was measured by varying the shear strain at 1% and 100% under a constant temperature of 15 °C.

### In‐Bath Coaxial Bioprinting of BV and LV

The BV and LV were produced using dual coaxial nozzles (ramé‐hart). Before printing, 40% w/v Pluronic F127 dissolved in 100 × 10^–3^ M CaCl_2_ solution (CPF127) was prepared as sacrificial ink and loaded into the core nozzle. HDMECs (1 × 10^7^ cells mL^−1^) and HDLECs (1 × 10^7^ cells mL^−1^) were encapsulated with pH‐neutralized hybrid bioink composed of 3% w/v VdECM bioink and 6% w/v sodium alginate. The bioink was loaded into the shell nozzle. Before in‐bath coaxial bioprinting, a housing structure was fabricated from PEVA, followed by deposition of the SdECM bath in the middle of the structure. Then, each cell was alternately printed into the bath on the designed printing paths. During the printing process, the extrusion of CPF127 from the core nozzle can facilitate the fabrication of the perfusable vessels and diffuse Ca^2+^ into the crosslinked alginate in the hybrid bioink. Subsequently, low‐melting‐point agarose (LMPA) was applied to the side chambers of the platform to mount the fabricated vessels. CPF127 was discarded during the gelation of the VdECM and SdECM bioinks at 37 °C for 30 min, and the platforms were cultured by circulating EGM‐MV and EGM‐MV2 in each channel using a rocking incubator.

### In‐Bath Bioprinting of Melanoma Spheroids

Porcine skin tissue, collected from a local slaughterhouse, was decellularized and formulated into a bioink following the steps listed in our previous studies.^[^ ^12^, ^34^ ^]^ The lyophilized tissues were dissolved in 0.5 M CH₃COOH solution containing 10 mg of pepsin per 100 mg of SdECM for seven days to produce the SdECM bioink. After the complete dissolution of SdECM, the pH of SdECM bioink was adjusted to 7.4 with a 10 M NaOH solution. Before printing, SK‐MEL‐28 and NHDFs‐Ad were encapsulated in 1% w/v SdECM bioink with a density of 10 × 10^7^ cells mL^−1^ and loaded in a sterile syringe connected to a 26G stainless steel microneedle. Meanwhile, 0.75% w/v SdECM bioink was placed in the PEVA (PolyScience) structure. A series of melanoma spheroids was gradually printed within the SdECM bath for ≈200 ms at a pressure of 7–13 kPa on a predefined printing path. The printing pressure was changed while maintaining a constant time deposition (≈200 ms) to vary the diameter of the spheroids. The printing needle was withdrawn after printing the spheroids, and the printed tissues were transferred into an incubator at 37 °C for cross‐linking.

### Transwell Coculture Assay

For transwell coculture assay, melanoma spheroids embedded in the SdECM bath were cultured on top of a filter and its lower well was seeded with dermal fibroblasts (Figure S3, Supporting Information). For comparison, a monoculture group, with only dermal fibroblasts seeded in the lower well, was prepared. After three days of coculturing, IF staining and qRT‐PCR was performed to observe the activation of fibroblasts in each group (see the list of primers in Table S1 in the Supporting Information). For the wound healing assay, the fibroblasts were cocultured with the melanoma spheroids for 72 h in 6‐well plates before the wounds were generated. Fibroblasts cultured in DMEM with 10% FBS were used as the control group. Next, each well was cultured with serum‐free DMEM for 72 h and imaged over this period. The area of the scratched field was measured using ImageJ, and each sample was assessed in three fields for replicates.

### Cancer Sprouting Assay

The representative confocal images were copied into ImageJ (NIH) to measure the invasion length and number of sprouting melanoma spheroids over time. The sprouts of the melanoma spheroids were defined against the background using the threshold function. The length of each sprout was calculated after eliminating the background. After conducting measurements on a group of samples, the recorded data were copied into SigmaPlot (Systat Software) for future analysis.

### IF Staining

For the IF staining experiments, the composition of the samples was fixed as 4% w/v paraformaldehyde solution for 2 h. After washing with 1 × PBS solution, the samples were permeabilized with 0.1% Triton X‐100 solution (Biosesang) for 15 min and treated with 1% w/v bovine serum albumin (BSA) for 30 min to block reactive epitopes. Primary antibodies were diluted in BSA and incubated with the samples at 4 °C overnight (see the list of antibodies in Table S2 in the Supporting Information). After gently washing with 1 × PBS, the secondary antibodies, Alexa Flour 488 and 594 were added to the sample and incubated for 2 h at room temperature. All samples were counterstained with DAPI (Thermo Fisher Scientific) for 1 h. The stained samples were imaged under a confocal microscope (LSM 900, Zeiss).

### Cell Viability Assay

The viability of the melanoma spheroids after drug administration was evaluated using a live/dead viability kit (Invitrogen). For the assay, the samples were washed with 1 × PBS and then transferred to the staining solution containing calcein AM (0.5 µl mL^−1^) and ethidium homodimer (2 µl mL^−1^, EthD‐1). After incubation at 37 °C for 30 min, the live/dead staining images were acquired using a confocal microscope. The cell viability was measured by calculating the ratio of viable cells to the total number of cells. The process was repeated to acquire cell viability data for the BV and LV. Nonfluorescent melanoma cells were used for the experiment to avoid wavelength interference.

### Permeability Test

For the quantitative comparison of endothelial disruption in BV and LV, FITC‐dextran (Merck) in EGM‐2 and EGM‐MV2 were perfused through BV and LV for 30 min. The diffusional profiles of FITC‐dextran were examined and recorded using a fluorescence microscope (Axio Zoom, Zeiss) every 5 min. ImageJ was used to analyze the mean fluorescence intensity from the entire region of each image. The diffusional permeability was calculated according to the following equation.

(1) $$P_{d}=\frac{1}{I_{i}-I_{b}}\;\left(\frac{I_{t}-I_{i}}{2}\right)\frac{d}{4}$$

where *P_d_* is the diffusional permeability coefficient, determined by initial fluorescent intensity (*I_i_*), background fluorescent intensity (*I_b_*), final intensity (*I_t_*) at some time (*t*), and vessel diameter (*d*)^[^ ^35^ ^]^

### Statistical Analysis

Quantitative values were presented as the mean of ± standard deviation and all experiments were performed in triplicate. For statistical analysis, a two‐tailed Student's *t*‐test was used to discriminate significant differences between the two experimental groups. For multiple experimental groups, statistical analysis was performed using one‐way ANOVA with Turkey's post hoc test. Differences were considered statistically when the *p*‐value was less than 0.05. Statistical analysis was carried out using SigmaPlot v12.5 (Systat Software, USA) Software.

## Conflict of Interest

The authors declare no conflict of interest.

## Supporting information

Supporting information

## Data Availability

The data that support the findings of this study are available from the corresponding author upon reasonable request.
