# Supplementary material for: Blood‐Lymphatic Integrated System with Heterogeneous Melanoma Spheroids via In‐Bath Three‐Dimensional Bioprinting for Modelling of Combinational Targeted Therapy
Source: Adv Sci (Weinh). 2022 Aug 26;9(29):2202093. doi: 10.1002/advs.202202093 (PMC9561777; doi:10.1002/advs.202202093)
Supplement: Supplementary file 1 — Supporting information [file ADVS-9-2202093-s001.pdf]

ADVANCED SCIENCE
Open Access

Supporting Information

for *Adv. Sci.*, DOI 10.1002/advs.202202093

Blood-Lymphatic Integrated System with Heterogeneous Melanoma Spheroids via In-Bath Three-Dimensional Bioprinting for Modelling of Combinational Targeted Therapy

Won-Woo Cho, Minjun Ahn, Byoung Soo Kim and Dong-Woo Cho**

Supporting Information

Blood-lymphatic integrated system with heterogeneous melanoma spheroids via in-bath three-dimensional bioprinting for modelling of combinational targeted therapy

Won-Woo Cho, Minjun Ahn, Byoung Soo Kim, Dong-Woo Cho**

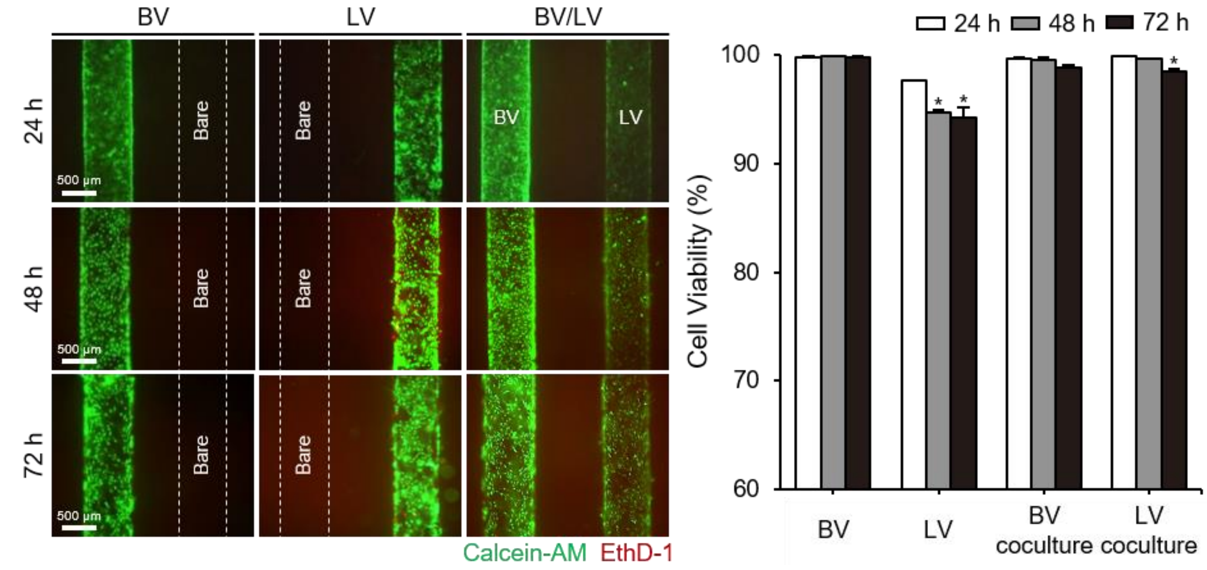

Figure S1. Live/dead assay of BV and LV. Live/dead staining to assess cell viability of BV and LV under monoculture and coculture conditions. Error bars indicate the SD (n = 3). *p*-values are calculated using one-way ANOVA with Turkey's post hoc test.

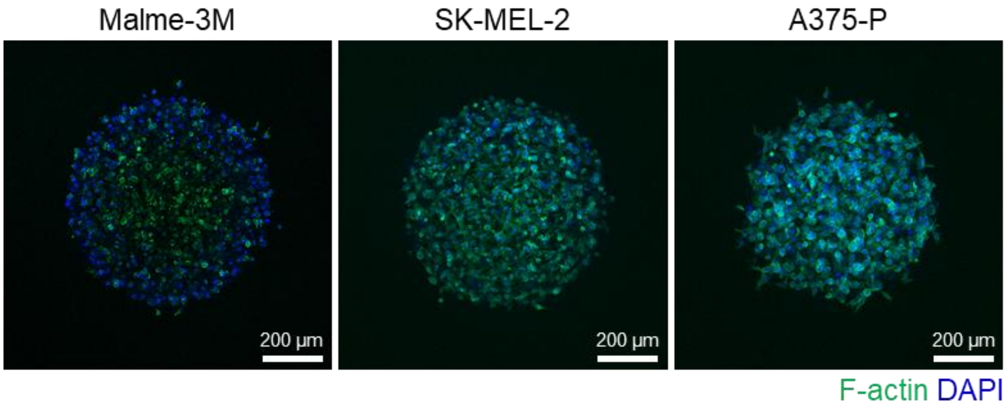

Figure S2. In-bath bioprinted melanoma spheroids using various metastatic melanoma cell lines (Malme-3M, SK-MEL-2, A375-P).

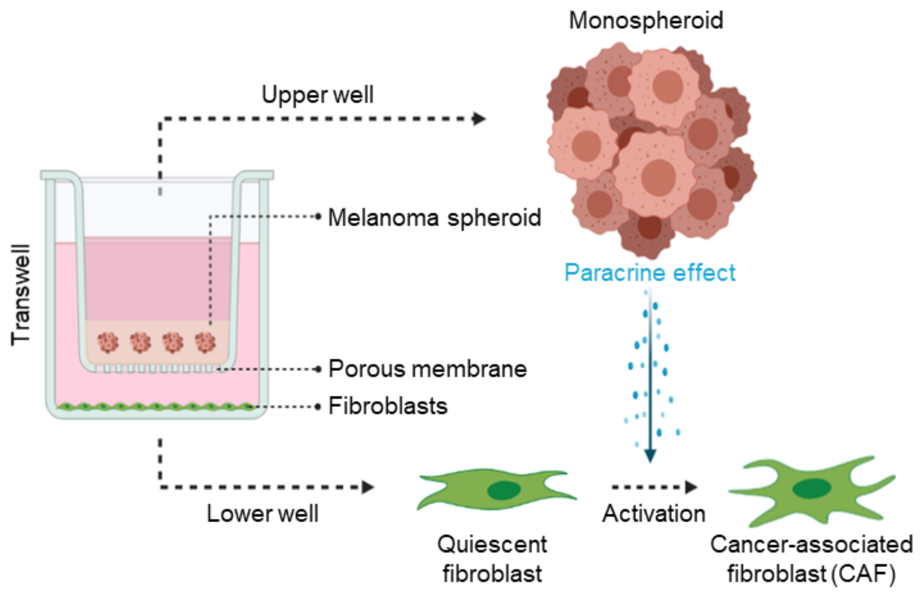

Figure S3. Coculture of fibroblasts and melanoma spheroids using transwell culture system. Transwell coculture system is used to study the paracrine effect of the melanoma spheroids on fibroblast activation. For the assay, the fibroblasts are culture on 6-well plates. Once confluent, the melanoma spheroids embedded within the SdECM bath are cultured in the upper well to observe the paracrine effect of the melanoma spheroids on fibroblast activation.

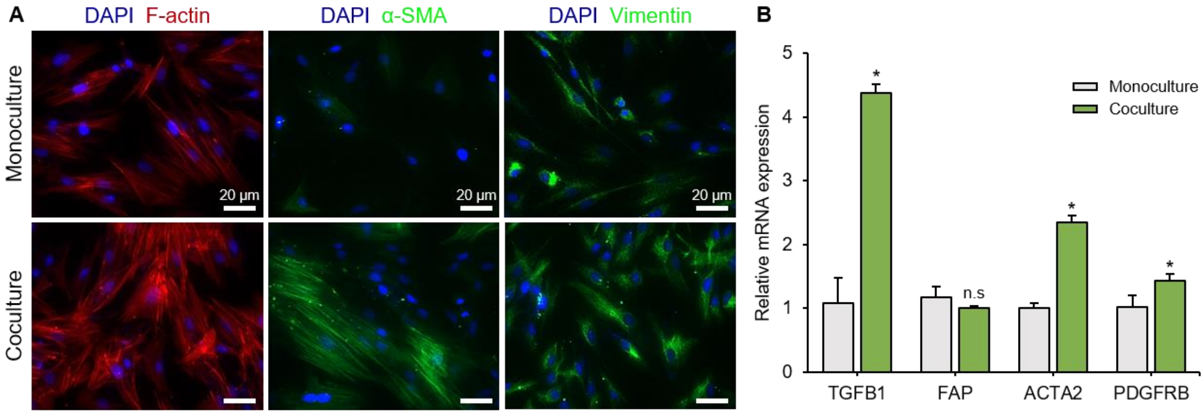

Figure S4. Fibroblast activation induced by paracrine signaling of melanoma spheroids. (A) IF staining images of the fibroblasts culture with (coculture) or without (monoculture) melanoma spheroids. **(B)** mRNA expression levels of TGFB1, FAP, ACTA2, and PDGFRB in the fibroblasts. Error bars indicate the SD (n = 3). $^*p < 0.05$ by a two-tailed Student's *t*-test.

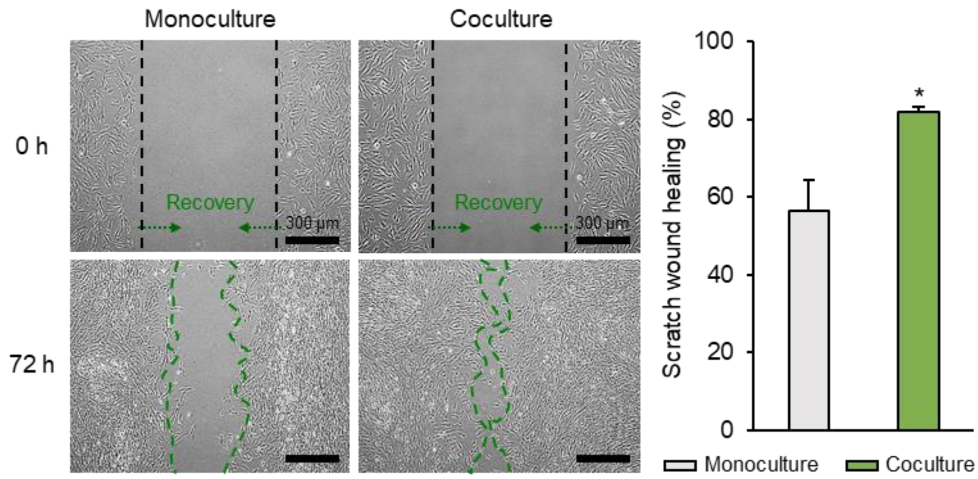

Figure S5. Wound healing assay to assess cell migration. Wound healing assay is performed over 72 h for the fibroblasts cultured with or without melanoma spheroids. The ratio of the recovered area (green dash line) to the original wound area (black dash line) is calculated. Error bars indicate the SD (n = 3). *p < 0.05 by a two-tailed Student's *t*-test.

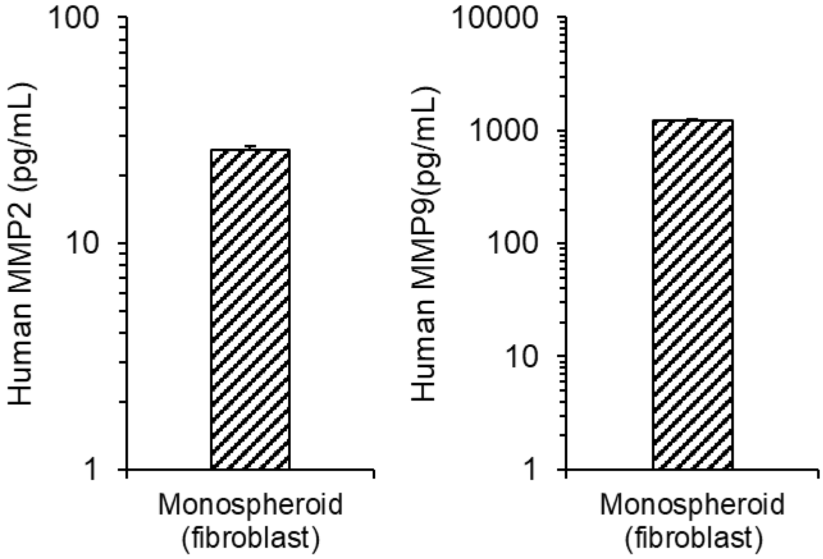

Figure S6. The amount of MMP-2 and MMP-9 secretion by the fibroblasts monospheroids on day 3. Error bars indicate the SD (n = 3).

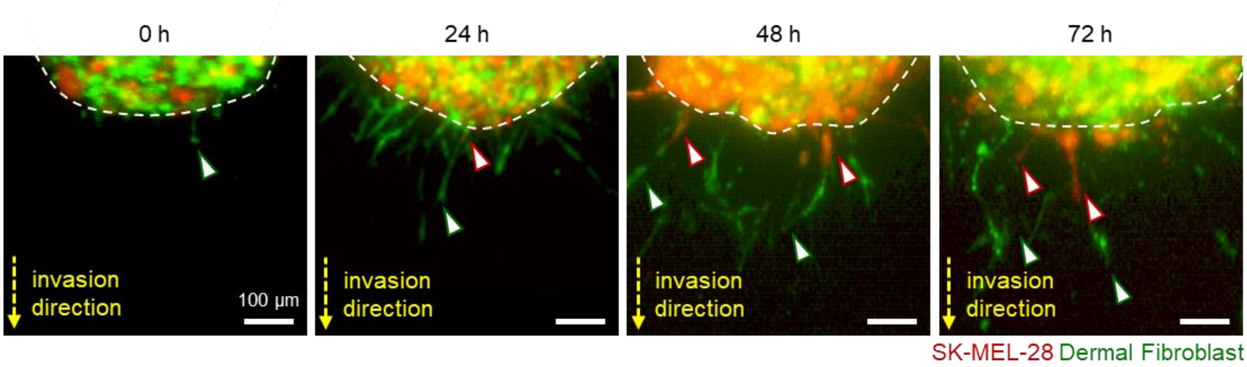

Figure S7. Confocal images of melanoma spheroids during 72 h of invasion. During *in vitro* culture, melanoma cells (red arrow) follow the migration path of the preceding fibroblasts, demonstrating fibroblasts-assisted melanoma invasion.

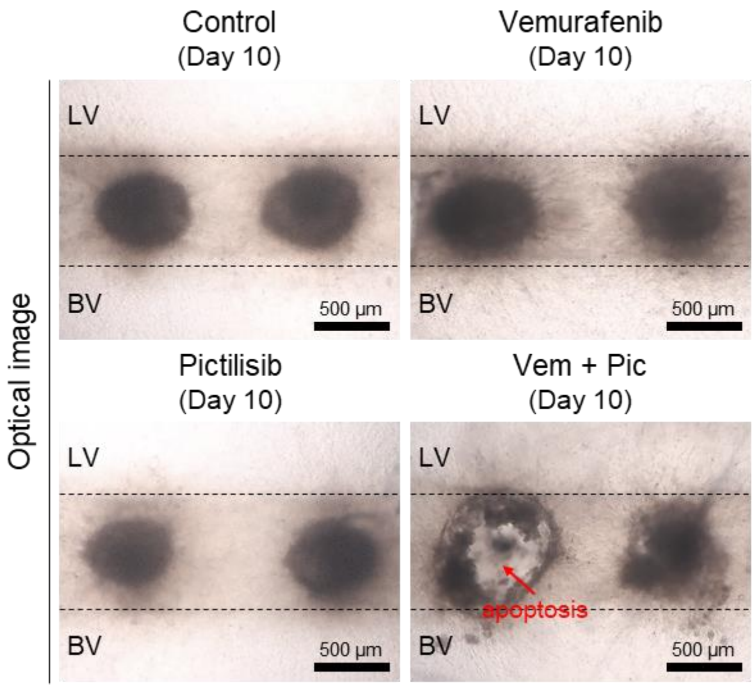

Figure S8. Optical images of melanoma spheroids after drug administration.

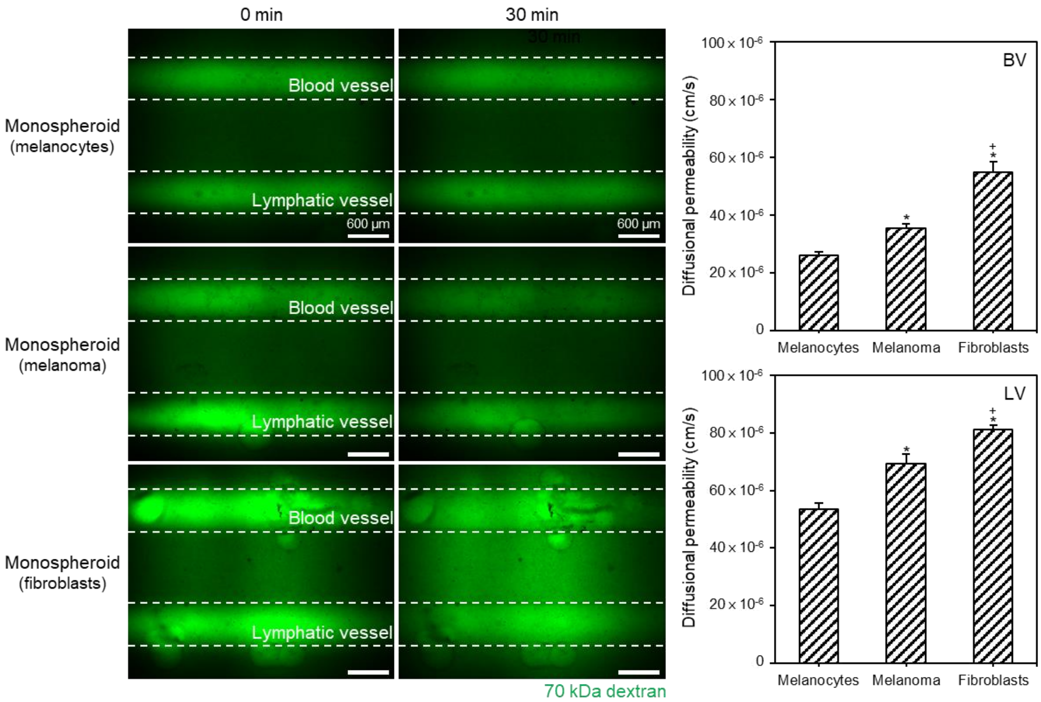

Figure S9. Diffusional permeability of BV and LV cultured with monospheroids made of normal melanocytes, melanoma cells, or fibroblasts. Error bars indicate the SD (n = 3). *p*-values are calculated using one-way ANOVA with Turkey's post hoc test. *$p < 0.05$ shows the significance between the melanocytes group and the rest. +$p < 0.05$ shows the significance between the melanoma group and the fibroblasts group.

Table S1. Sequences of primers used in this experiment.

Primer	Primer sequence (5'→3')
TGFB1	F: TACCTGAACCCGTGTTGCTCTC<br>R: GTTGCTGAGGTATCGCCAGGAA
FAP	F: GGAAGTGCCTGTTCCAGCAATG<br>R: TGTCTGCCAGTCTTCCCTGAAG
ACTA2	F: CTATGCCTCTGGACGCACAACT<br>R: CAGATCCAGACGCATGATGGCA
PDGFRB	F: TGCAGACATCGAGTCCTCCAAC<br>R: GCTTAGCACTGGAGACTCGTTG
GAPDH	F: GTCTCCTCTGACTTCAACAGCG<br>R: ACCACCCTGTTGCTGTAGCCAA

Table S2. Lists of antibodies used in this experiment.

Antibody	Company	Catalog Number	Dilution
CD31	Abcam	ab9498	1:500
ZO-1	ThermoFisher	40-2200	1:400
LYVE1	Abcam	ab33682	1:200
PROX1	Abcam	ab101851	1:200
VE-cadherin	ThermoFisher	14-1441-82	1:100
α-SMA	Abcam	ab7817	1:100
Fibronectin	Abcam	ab2413	1:250
MCL1	ThermoFisher	MA5-15236	1:200
Ki-67	ThermoFisher	14-5699-82	1:100
Vimentin	Abcam	ab8978	1:100
